# Weak association between the interleukin-8 rs4073 polymorphism and acute pancreatitis: a cumulative meta-analysis

**DOI:** 10.1186/s12881-019-0861-4

**Published:** 2019-07-24

**Authors:** Yening Li, Jing Bai, Bing He, Nan Wang, Haoran Wang, Dongliang Liu

**Affiliations:** Cardiovascular Institute of Luohe and Departments of Intense Care Unit (YL), Endocrinology (JB), Arthritis Surgery (BH), Pediatrics (NW) and Cardiology (HW and DL), Luohe Central Hospital, Luohe Medical College, 56# Renmin Ave, Luohe, 462000 People’s Republic of China

**Keywords:** Interleukin, Gene polymorphisms, Acute pancreatitis, Meta-analysis, Interleukin-8, Single nucleotide polymorphism

## Abstract

**Background:**

Several studies have been performed to investigate the associations between interleukin (IL)-8 rs4073 polymorphism and acute pancreatitis (AP), but the results are inconclusive. We conducted this cumulative meta-analysis for a precise estimate of the relationship between IL-8 rs4073 polymorphism and acute pancreatitis.

**Methods:**

We searched the electronic databases for relevant studies. Odds ratios (ORs) and 95% confidence intervals (CIs) were calculated to evaluate the strength of the associations. For a better presentation of how the pooled ORs changed as updated evidence accumulated, we used forest plots from a cumulative meta-analysis method.

**Results:**

Ten studies involving 1646 AP patients and 1816 controls were finally included in this meta-analysis. Cumulative meta-analyses indicated there is a consistent trend toward association after the initial discovery. Under the allelic, dominant, recessive and homozygous models, the pooled ORs were 1.265 (1.147–1.395, *p* < 0.001), 1.304 (1.127–1.508, *p* < 0.001), 1.431 (1.203–1.702, *p* < 0.001), and 1.634 (1.334–2.001, *p* < 0.001), respectively.

**Conclusions:**

This meta-analysis demonstrated a suggestive result that people who carried the risk A allele of the IL-8 rs4073 polymorphism may be more sensitive to acute pancreatitis.

**Electronic supplementary material:**

The online version of this article (10.1186/s12881-019-0861-4) contains supplementary material, which is available to authorized users.

## Background

Acute pancreatitis (AP) is a relatively common disease. While most cases are mild, a significant fraction of patients suffers a severe course, resulting in systemic inflammatory response, multiple organ failure and prolonged hospitalization, and the mortality is very high despite intensive care [1, 2]. Therefore, early detection of patients at risk of AP is important for reducing adverse events and improving the outcomes. The pathophysiological progress involves a local and systematic inflammatory response to the autodigestion of the pancreatic tissue, and inflammation plays an integral role [3]. Because the mortality in AP is closely associated with an uncontrolled inflammation, and an excessive secretion of cytokines is essential in the inflammatory process, it is reasonable to hypothesis that genetic variants in the cytokine genes may play a role in the risk of AP.

Among all the cytokines, interleukin (IL)-8 stands out in the AP pathophysiology as it has been demonstrated to be significantly elevated during the pathological course of AP, and the level was reported to be associated with the severity of AP [4–6]. A functional single nucleotide polymorphism (SNP) in the IL-8 promotor (− 251 T/A, rs4073) has been implicated in several inflammatory conditions [7–9]. But it’s role in AP is still controversial according to the current evidence. Due to the contradictory and inconclusive results, we performed this meta-analysis to clarify the correlation between IL-8 rs4073 polymorphism and AP risk.

## Methods

The current study was performed following Preferred reporting items for systematic review and meta-analysis (PRISMA) guidelines for the development of protocols [10] and reporting all the necessary items [11].

### Search strategy

All studies reporting the relationships of IL-8 polymorphisms with AP published before March 30, 2019 were identified by computerized searches in databases including “Pubmed”, “Embase”, “China National Knowledge Infrastructure (CNKI)”, and “Wanfang”. The search terms were: (“interleukin-8” OR “IL-8”) AND (“gene” OR “polymorphism” OR “genetic variant”) AND (“acute pancreatitis” OR “pancreatitis”). In addition, we checked the references of all the retrieved articles to identify further relevant articles.

### Inclusion and exclusion criteria

Qualified studies have to meet the following criteria: (1) predefined diagnosis criteria for AP; (2) case-control studies evaluated the association between the IL-8 rs4073 polymorphism and AP risk; (3) provided detailed genotype frequencies; (4) if multiple reports based on the same study population were available, the most recent or largest study was selected. The main exclusion criteria were: (1) insufficient data offered to calculate OR estimate; (2) non-case-control studies; (3) duplicate data from a same cohort. We screened titles and abstracts of searching results for an initial evaluation, and full articles were further evaluated to confirm eligibility.

### Data extraction

All data were extracted independently by two reviewers (Y.L. and J.B.). The following information was extracted or calculated from the included articles: authors, publication year, country, ethnicity, Hardy-Weinberg equilibrium (HWE) status, genotyping method, the number of cases and controls, and counts of alleles and genotypes in both cases and controls. The results were compared, and differences were resolved by discussion and consensus with a third reviewer (H.W.).

### Quality assessment

Two authors (B.H. and N.W.) independently assessed the quality of the included studies based on Newcastle Ottawa Scale (NOS) [12]. This scale uses a star rating system to judge the methodological quality and consists of three parts: selection, comparability and ascertainment of exposure. A full score is 9 stars, and a score of five or more was regarded as “high quality”; otherwise, the study was regarded as “low quality”. Any disagreements on the NOS score of the studies were resolved through a comprehensive reassessment by the other authors.

### Statistical analysis

Crude Odds ratio (ORs) with corresponding 95% confidence intervals (CIs) were used to estimate the strength of association between the IL-8 rs4073 polymorphism and AP risk. The statistical significance of the pooled ORs was examined by Z test and *P* < 0.05 was considered statistically significant. In the meta-analysis, the overall pooled ORs were calculated for four models, including the allelic, dominant, recessive and homozygous models. Deviation from Hardy-Weinberg equilibrium (HWE) was examined by Chi-square test and *P* value < 0.05 indicated a departure from HWE. The between-study heterogeneity was assessed by the I^2^ statistic, which was calculated from the Q statistic [13]. If the heterogeneity was statistically significant (*P* < 0.05 for Q-test or I^2^ > 50%), the random-effects model (based on DerSimonian-Laird method) was adopted to get the pooled estimates [14]; otherwise, the fixed-effects model (based on Mantel-Haenszel method) was used [15]. To reduce the risk of false positive when performing repetitive estimates, the cumulative meta-analysis method was adopted [16]. To assess the stability and reliability of the meta-analyses, we performed sensitivity analyses by sequentially omitting 1 estimate at a time and recalculating the pooled results. The results were suggested to be robust if any single study didn’t alter the significance of our results. Potential publication bias was assessed with Begger’s funnel plot and the Egger’ linear regression test, and a *P* < 0.05 was considered significant [17, 18]. The statistical analysis was performed with STATA statistical software (Version 13.3; Stata Corporation, College Station, TX, USA). All *P* values were two-tailed.

## Result

### Characteristics of included studies

**Fig. 1:**
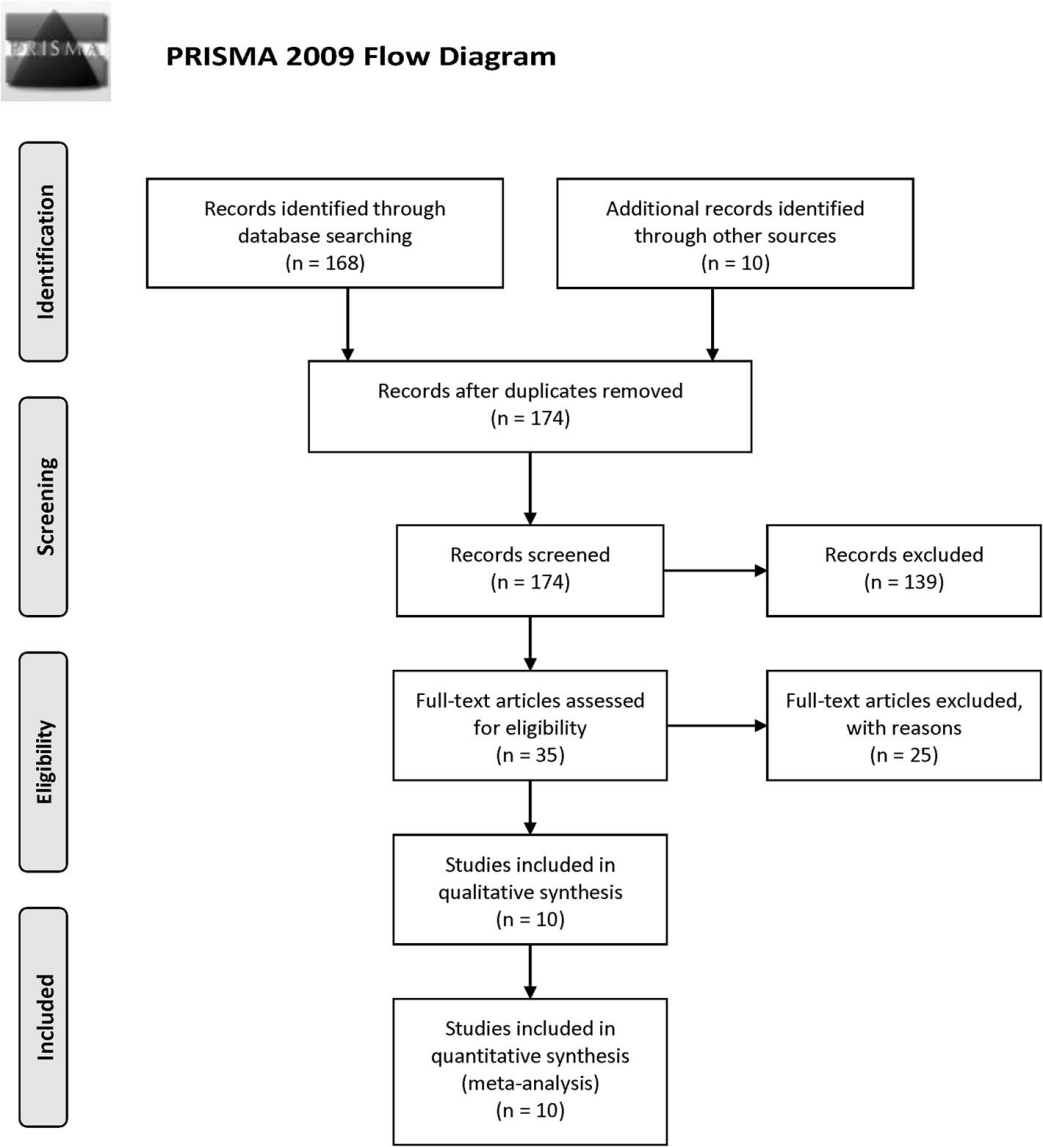
PRISMA Flow-diagram depicting identification and selection process for the present meta-analysis

Our literature screening process is shown in Fig. 1. In the initial screening process, a total of 178 articles were retrieved based on our selection strategy from the databases. After the removal of duplicated literatures and articles that did not meet the inclusion criteria, 10 studies involving 1646 AP patients and 1816 controls were finally included in this meta-analysis [19–28]. Of these selected studies, six studies were published in English [19–21, 24–26], three were published in Chinese [22, 27, 28], the other one was published in Russian [23]. There were six studies conducted in China [20, 22, 24, 26–28], and one study conducted in U.S. [21], Russia [23], Turkey [19] and Hungary [25], respectively. Table 1 summarizes the basic information of each study, including the year of publication, country, ethnicity, sample size, NOS score and so on.

**Table 1:** Characteristics of included studies

Author, year	Language	Country	Ethnicity	Sample size (M/F)		Age		Genotype method	NOS score
				Case	Control	Case	Control		
Hofner et al., 2006	English	Hungary	Caucasion	92(NA/NA)	200(NA/NA)	NA	NA	PCR	5
Li et al., 2007	Chinese	China	Asian	71 (33/38)	70 (39/31)	46.3 ± 14.3	48.9 ± 15.1	PCR	4
Chen and Nie, 2008	English	China	Asian	101 (60/41)	120 (71/49)	51.57 ± 13.39	51.05 ± 9.37	PCR	6
Cao and Xiao, 2010	Chinese	China	Asian	119 (57/62)	236 (130/106)	46.1 ± 13.8	38.8 ± 6.7	PCR	4
Tang et al., 2010	Chinese	China	Asian	170(NA/NA)	132 (65/67)	NA	NA	PCR	4
Chantsev and Leonov, 2014	Russian	Russia	Caucasion	100(NA/NA)	100(NA/NA)	NA	NA	PCR	4
Li et al., 2015	English	China	Asian	176 (115/61)	176 (115/61)	51.6 ± 11.3	52.3 ± 10.9	PCR	6
Bao et al., 2015	English	China	Asian	335 (205/130)	335 (205/130)	NA	NA	PCR	6
Anilir et al., 2017	English	Turkey	Caucasion	176(NA/NA)	100(NA/NA)	NA	NA	PCR	6
Bishu et al., 2018	English	USA	Mixed	357 (178/179)	347 (135/212)	52 ± 19	54 ± NA	PCR	6

### Meta-analysis results and heterogeneity analysis

**Fig. 2:**
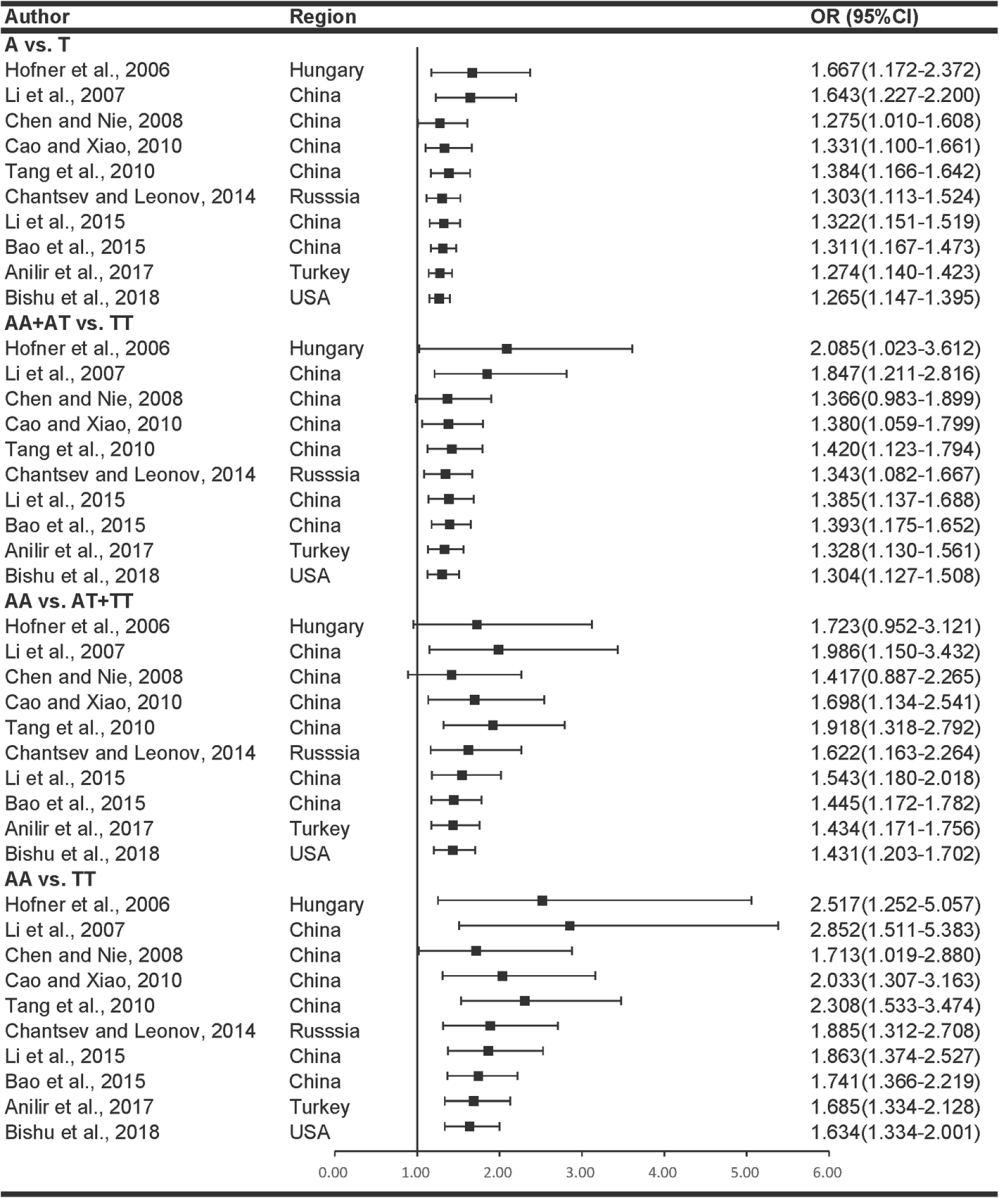
Cumulative meta-analysis describing the association between IL-8 rs4073 variant and AP in four genetic models. The results indicated there is a consistent trend toward association after the initial discovery

Meta-analyses under four genetic models are shown in Fig. 1. When comparing effect sizes, we found no evidence of significant heterogeneity in the analyses of all the four genetic models used, thus fixed-effects models were used. Cumulative meta-analyses (Fig. 2) indicated there is a consistent trend toward association after the initial discovery. The pooled results showed that the IL-8 rs4073 polymorphism associates with an increased risk of AP. Under the allelic, dominant, recessive, and homozygote models the ORs, 95% CIs and its *p*-values were 1.265 (1.147–1.395, *p* < 0.001), 1.304 (1.127–1.508, *p* < 0.001), 1.431 (1.203–1.702, *p* < 0.001), and 1.634 (1.334–2.001, *p* < 0.001), respectively.

**Fig. 3:**
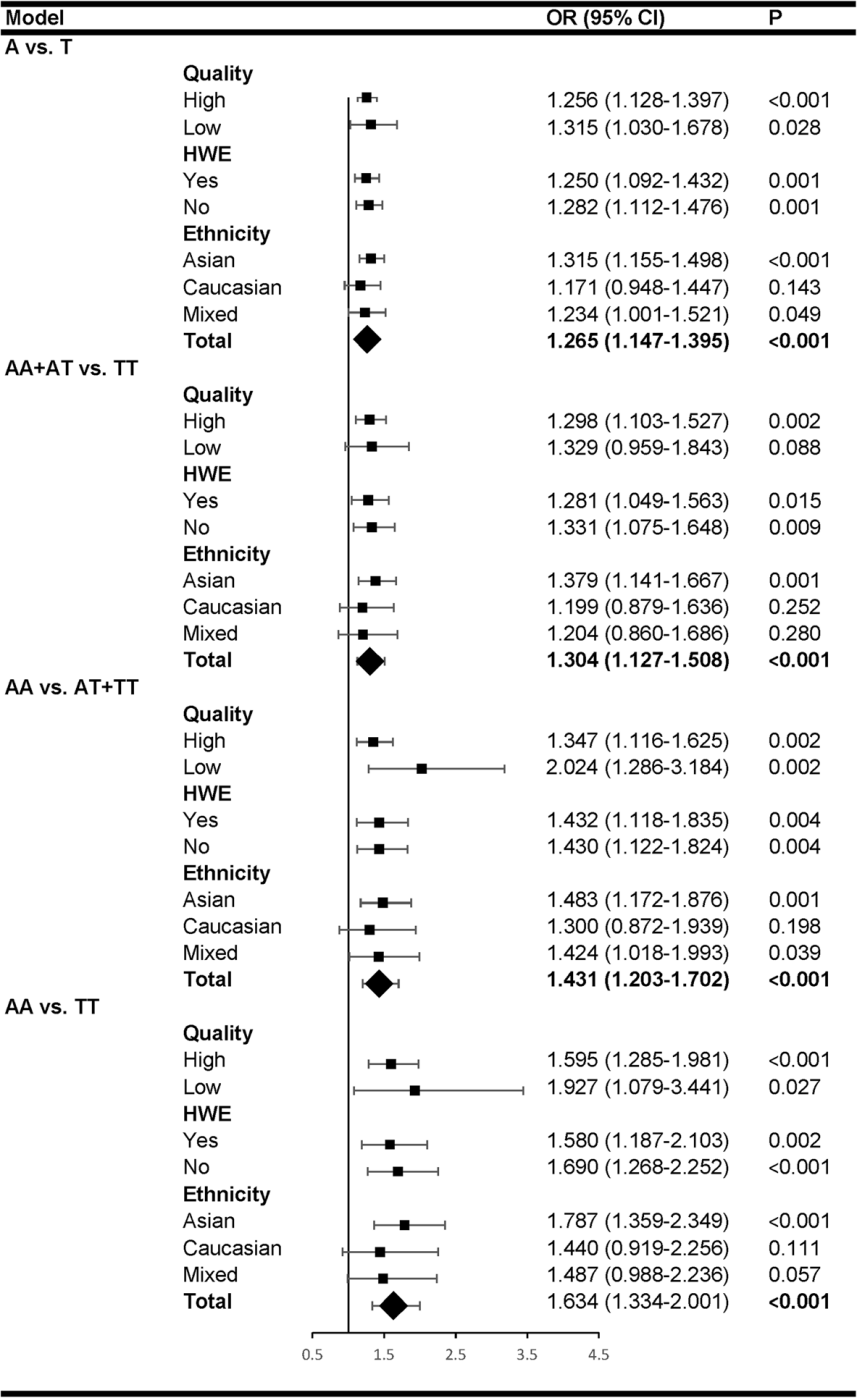
Subgroup analyses by study quality (high or low), HWE test results (yes or no) and ethnicity (Asian, Caucasian and mixed) in all the four genetic models. Trends toward an association between the IL-8 rs4073 polymorphism an AP risk were observed in all the subgroups, and statistically significant association was found in most subgroups

Subgroup analyses (Fig. 3) were performed according to the study quality (high or low), HWE test results (yes or no) and ethnicity (Asian, Caucasian or mixed) in all the four genetic models. Trends toward an association between the IL-8 rs4073 polymorphism an AP risk were observed in all the subgroups, and statistically significant association was found in most subgroups.

### Sensitivity analysis and publication bias

**Fig. 4:**
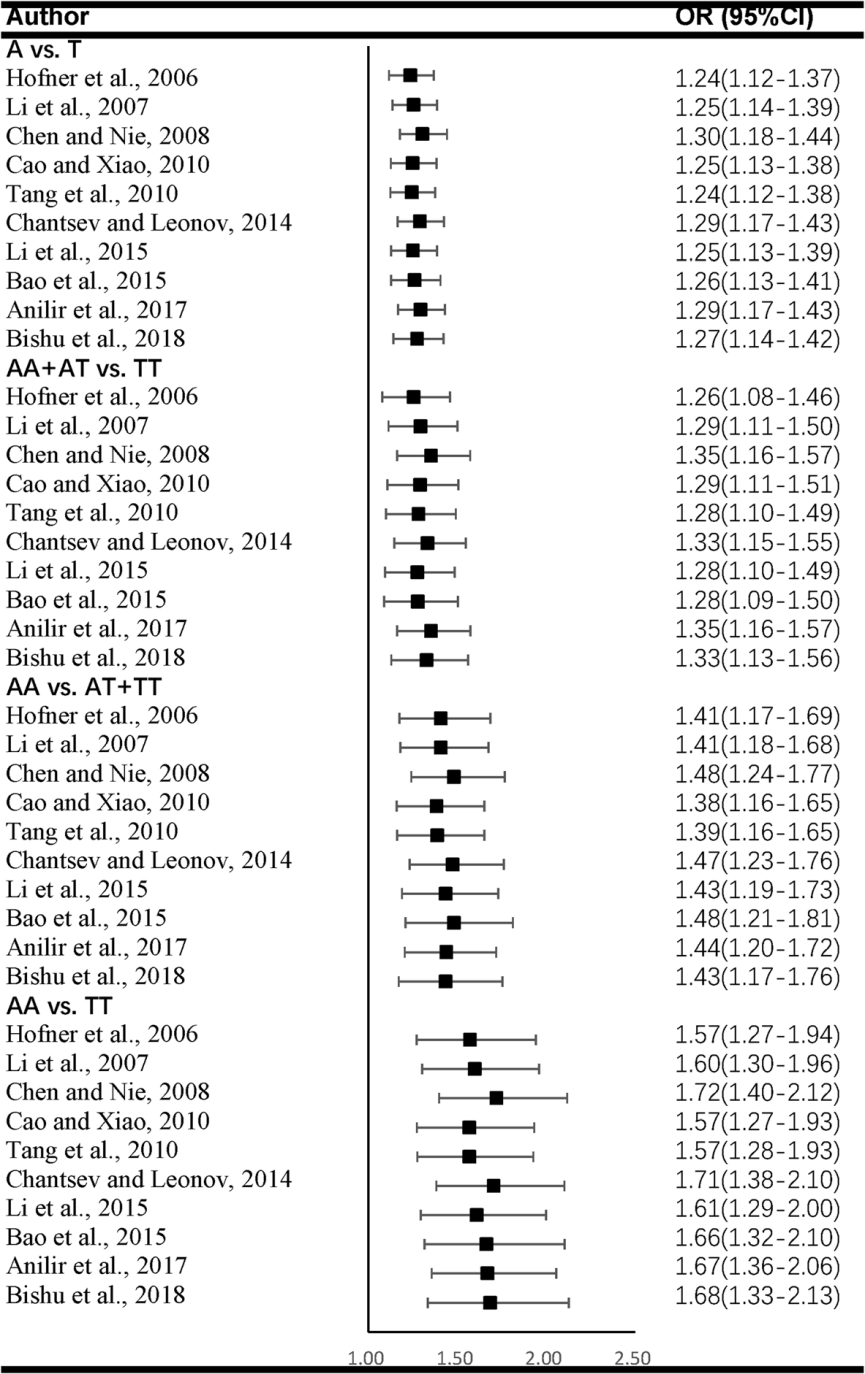
Sensitivity analyses of all the four genetic models by omitting one study at a time

We estimated the stability of all the pooled ORs by eliminating all included studies one by one. The corresponding ORs were not statistically altered, indicating that our results were stable. (Fig. 4).

**Fig. 5:**
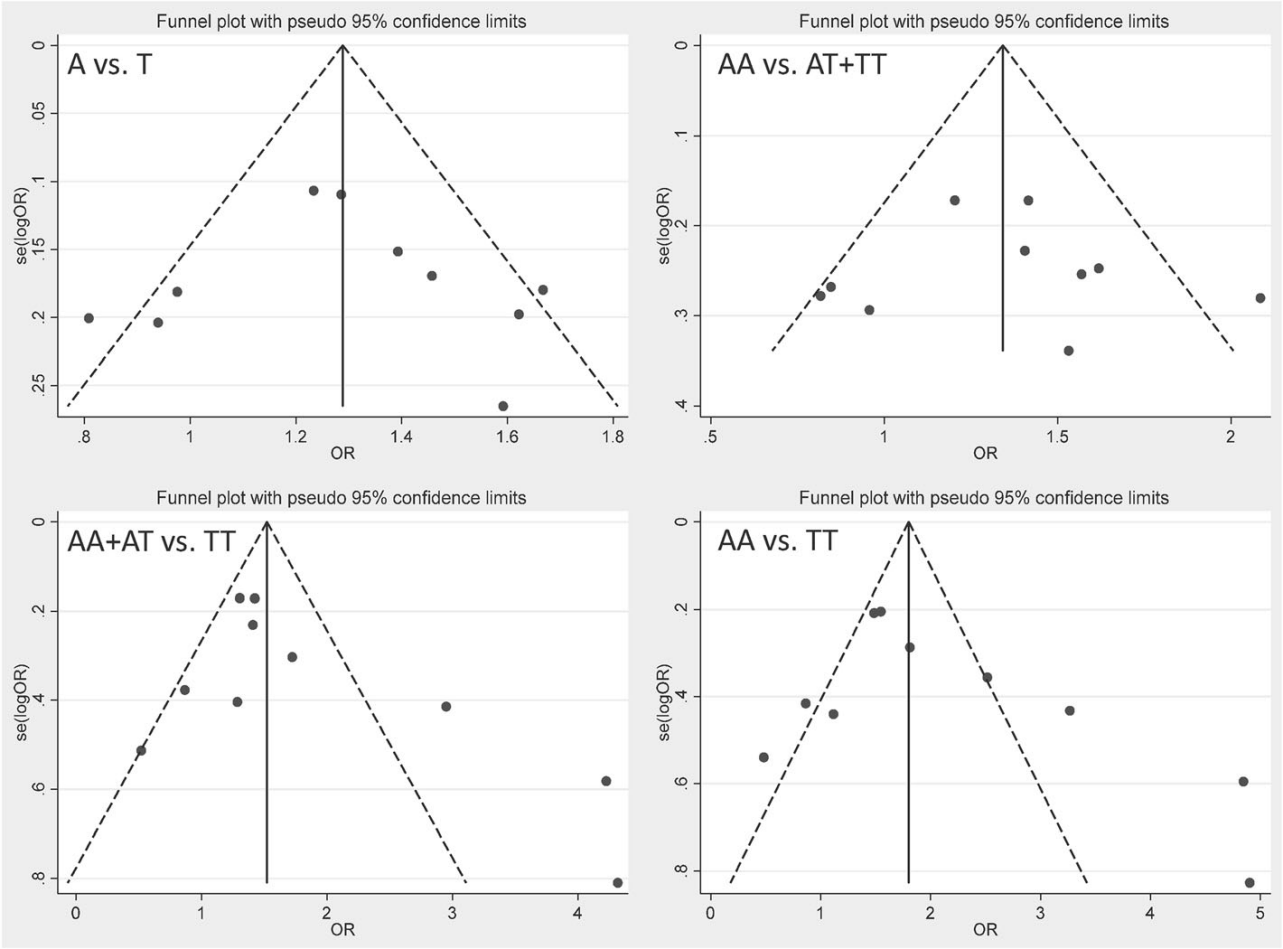
Begger’s funnel plots to evaluate publication bias. The shape of the funnel plot was obviously symmetrical for all genetic models

Egger’s linear regression tests and funnel plots were performed to assess possible publication bias in the meta-analyses. The shape of the funnel plot was obviously symmetrical for all models (Fig. 5). The *P* value of Egger’s test also suggested that there was no significant publication bias (Table 2).

**Table 2:** Detailed results of Egger’s test

Model	t	*p*
A vs. T	0.17	0.872
AA vs. AT+TT	0.07	0.948
AA+AT vs. TT	1.82	0.106
AA vs. TT	1.63	0.143

## Discussion

AP is a multifactorial disorder which could be attributed to both genetic and environmental factors. The association between the IL-8 rs4073 polymorphism and AP susceptibility has been studied for several years, however, the results are still controversial. As previous studies were limited by the small sample size, here we performed this meta-analysis to clarify the association by incorporating all available data. Cumulative meta-analysis showed that there is reliable evidence that the SNP is associated with increased risk of AP, and further similar studies are unlikely to change the current conclusion. If a future study reports a contrary result, adding its results into the pooled analyses will substantially increase the heterogeneity and reduce the weight it receives in the meta-analysis. As a result, future studies are unlikely to alter the current conclusion [16]. Further subgroup analyses stratified by study quality, HWE status and ethnicity indicated that the association was consistent across subgroups.

Detailed mechanisms underlying the observed association between IL-8 rs4073 polymorphism and AP is not clear. However, there were evidence that SNPs in the promotor region of IL-8 are able to modulate the IL-8 levels at both transcriptional and translational levels. The IL-8 rs4073 A allele, which is the risk allele of AP in the current study, is reported to be associated with increased IL-8 production [8]. IL-8 is now known to be an important mediator in many inflammatory diseases. And in AP patients, the levels of IL-8 are significantly elevated during the course of disease, and may serve as a predictive marker of severe AP [4–6]. The biological properties of IL-8 are similar to those of the classical chemokines [29, 30]. It has a C-terminal heparin-binding site that allows it to become immobilized within the endothelial. Therefore, it is reasonable to speculate that the genetic polymorphism affects the transcriptional activity of the promotor region of the IL-8 gene, regulates the expression level of IL-8 during the inflammatory process, and eventually modifies the risk and severity of AP in the population.

Key strengths of the current study are the comprehensive analyses, which were based on more studies, participants and AP patients compared to the previous analyses. Therefore, the statistical analyses have greater power, the estimates have more accuracy, and we were able to perform more detailed sub-group analyses. Besides, we adopted the cumulative meta-analyses by which we could assess the possibility of future studies on this topic to change the current conclusions.

When interpreting the results, several potential limitations in this meta-analysis should be considered. First, the sample of published studies remains small for a comprehensive analysis. Second, the source of articles is uneven in geographical distribution, the majority of the included studies were conducted in Asians which may introduce ethnicity bias, and further studies should focus on Africans and Caucasians. Third, the overall results of our study were based on individual unadjusted ORs because we had no access to the original data.

## Conclusions

In conclusion, this cumulative meta-analysis demonstrated a suggestive result that people who carried the risk A allele of the IL-8 rs4073 polymorphism may be more sensitive to acute pancreatitis. Furthermore, well-designed and larger studies, dealing specifically with gene-gene and gene-environment interactions, are warranted.

## Additional files

Additional file 1: **Table S1**. PRISMA-2009-Checklist. (DOC 65 kb)

Additional file 2: **Table S2**. Newcastle Ottawa Scale for quality assessment. (PDF 259 kb)

## Data Availability

All data generated or analysed during this study are included in this article [and its Additional files 1 and 2].

## Abbreviations

AP: Acute pancreatitis
CI: Confidence interval
CNKI: China National Knowledge Infrastructure
HWE: Hardy-Weinberg equilibrium
IL: Interleukin
NOS: Newcastle Ottawa Scale
OR: Odds ratio
PRISMA: Preferred reporting items for systematic review and meta-analysis
SNP: Single nucleotide polymorphism
