# Supplementary material for: Weak association between the interleukin-8 rs4073 polymorphism and acute pancreatitis: a cumulative meta-analysis
Source: BMC Med Genet. 2019 Jul 24;20:129. doi: 10.1186/s12881-019-0861-4 (PMC6657145; doi:10.1186/s12881-019-0861-4)
Supplement: Supplementary file 2 — Table S2. Newcastle Ottawa Scale for quality assessment. (PDF 259 kb) [file 12881_2019_861_MOESM2_ESM.pdf]

NEWCASTLE - OTTAWA QUALITY ASSESSMENT SCALE	Bishu et al., 2018	Anilir et al., 2017	Li et al., 2015	Chantsev and Leonov, 2014	Chen and Nie, 2008	Hofner et al., 2006	Cao and Xiao, 2010	Tang et al., 2010	Li et al., 2007	Bao et al., 2015
**Selection**										
1) Is the case definition adequate?										
a) yes, with independent validation ★	★	★	★	★	★	★	★	★	★	★
b) no description										
2) Representativeness of the cases										
a) consecutive or obviously representative series of cases ★	★	★	★		★	★				★
b) potential for selection biases or not stated										
3) Selection of Controls										
a) community controls ★	★	★	★		★	★	★	★		★
b) hospital controls										
c) no description										
4) Definition of Controls										
a) no history of disease (endpoint) ★	★	★	★	★	★				★	★
b) no description of source										
**Comparability**										
1) Comparability of cases and controls on the basis of the design or analysis										
a) study controls for confounding factors ★										
b) no adjustment										
**Exposure**										
1) Ascertainment of exposure										
a) reports genotype methods ★	★	★	★	★	★	★	★	★	★	★
b) no description										
2) Same method of ascertainment for cases and controls										
a) yes ★	★	★	★	★	★	★	★	★	★	★
b) no										
Total stars	6	6	6	4	6	5	4	4	4	6
